# Artificial-intelligence- and multi-omics-guided predictive and drug repurposing framework construction for colorectal cancer: evidence from succinylation–neutrophil signatures

**DOI:** 10.3389/fcell.2026.1808514

**Published:** 2026-09-03

**Authors:** Huimin Jie, Huaying Huo, Jiamei Wang

**Affiliations:** Shanxi Provincial People’s Hospital, Fifth Clinical Medical College, Shanxi Medical University, Taiyuan, Shanxi, China

**Keywords:** artificial intelligence, multi-omics, neutrophils, predictive model, succinylation, therapeutic framework

## Abstract

**Background:**

Succinylation-linked metabolic rewiring and neutrophil-driven inflammation are key drivers of colorectal carcinoma (COAD) progression; however, they have rarely been translated to an end-to-end artificial intelligence (AI) drug-design pipeline connecting target nomination, resistance-relevant tumor microenvironment states, and candidate evaluation.

**Methods:**

We integrated bulk transcriptomes from The Cancer Genome Atlas colon adenocarcinoma (TCGA-COAD) and Gene Expression Omnibus databases to derive succinylation–neutrophil (SN)-associated genes using the limma package, single-sample gene-set enrichment analysis, weighted gene coexpression network analysis, and protein–protein interaction network analysis; we then constructed a cross-cohort prognostic system by screening and selecting the machine-learning combination of random survival forest and ridge regression as the optimal predictor. To enable precision stratification, we applied deep-learning self-organizing maps (SOMs) to define the SN-driven molecular subtypes and characterized their immune and pathway heterogeneities at both bulk and single-cell resolutions through AI-powered virtual perturbation analysis to interrogate the cell-state shifts. Importantly, we extended AI approaches in drug design beyond risk modeling to a target-to-candidate workflow by nominating a druggable SN hub gene. We also performed co-culture *in vitro* assays for identifying NOX4 molecular insights into neutrophil patterns and their associations with COAD progression.

**Results:**

Our approach identifies SN biology as a clinically actionable axis in COAD, highlights NOX4 as a central SN-associated target with immune relevance, and prioritizes talazoparib as a repurposing candidate supported by integrated *in silico* evidence.

**Conclusion:**

The findings of this study provide a precision-oncology blueprint that couples AI stratification with therapeutic nomination and evaluation.

## 1 Introduction

Colorectal carcinoma (COAD) is a type of colorectal malignant tumor whose incidence and mortality rate have been increasing steadily over the years (Bell and Pai, 2022). This disease is characterized by significant heterogeneity, which complicates treatment outcomes and increases the risk of resistance to current therapies, such as surgery, chemotherapy, and radiotherapy (Benedict et al., 2015). As key players in the immune response, neutrophils produce a range of proinflammatory cytokines and reactive oxygen species (ROS) that may facilitate tumor progression and inflammation (Chiang et al., 2024). For example, the formation of neutrophil extracellular traps (NETs) can facilitate COAD progression (Kong et al., 2023). Moreover, single-cell analysis has revealed that abnormal accumulation of neutrophils in the COAD tumor microenvironment (TME) can lead to progression of COAD (Marteau et al., 2026). Succinylation is a post-translational modification (PTM) that alters the expression of cariogenic proteins to bridge the gap between the tumor metabolic and immune microenvironments (Hu et al., 2023; Wu et al., 2025). For example, succinylation can modify pathogenic protein expression, thereby promoting COAD progression and its TME (Jiang et al., 2025). Indeed, succinylation can lead to the activation of neutrophils and release of NETs (Barnes et al., 2011). In addition, succinylation in neutrophils has been implicated in the progression of various types of cancers (Shen et al., 2023). However, there is no evidence that succinylation modification of the neutrophilic protein contributes to the heterogeneity and progression of COAD. Hence, elaborating the mechanisms of succinylation modifications in neutrophils can provide additional insights into the pathogenesis of COAD. Advancements in artificial intelligence (AI) and multi-omics research can be used to elucidate the protein PTMs in cancer research systemically. For example, an AI-enabled independent study revealed that isonicotinylation can be considered as a predictive and multitarget framework for hepatocellular carcinoma patients (Zhou et al., 2026). In addition, AI and multi-omics pipelines have enabled the recognition that lactylation can also be considered as the prognostic and therapeutic target in colorectal cancer (Huang et al., 2024a).

**FIGURE 1:**
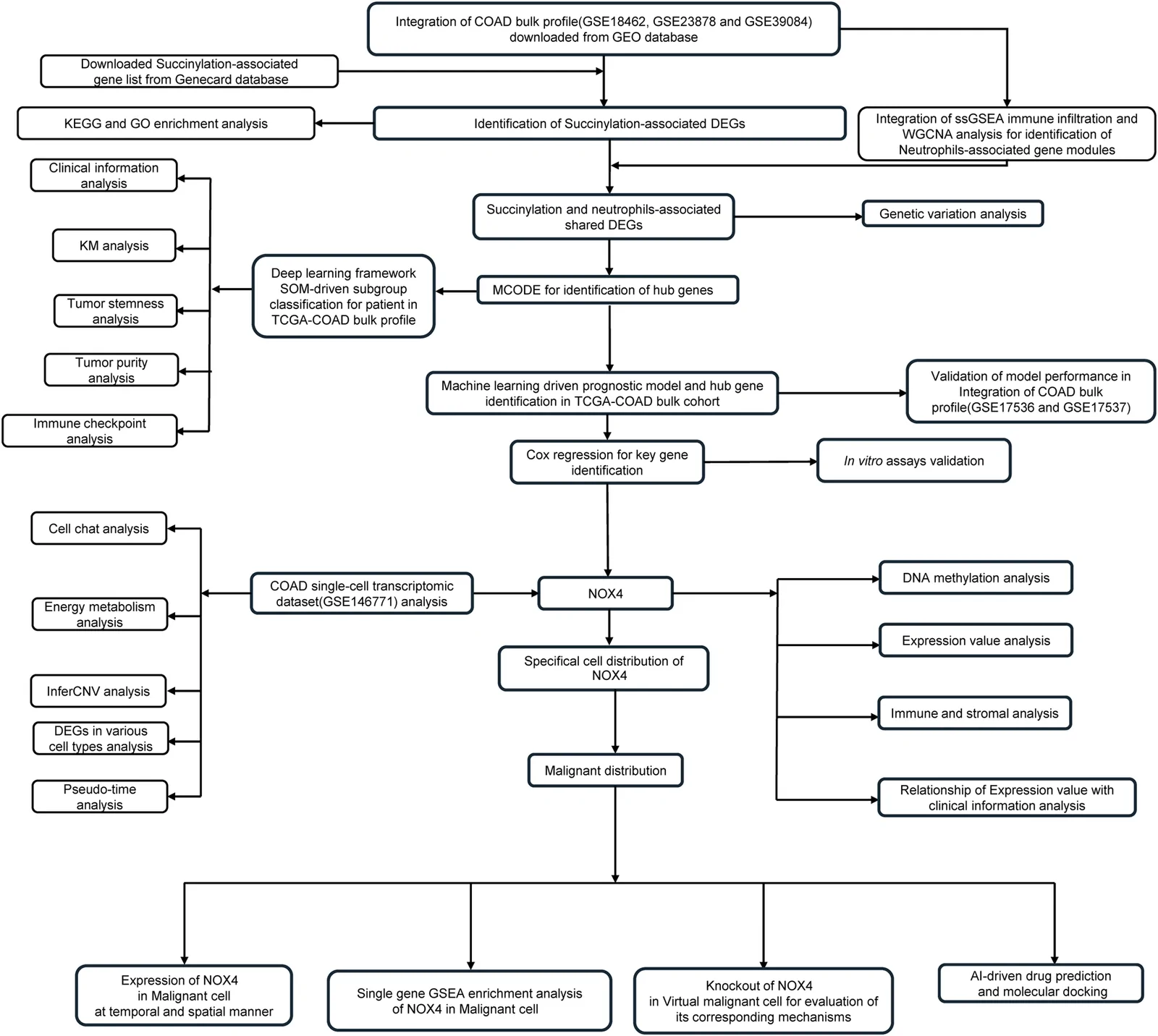
Workflow of the present study.

Overall, we first identified succinylation–neutrophil (SN)-associated differentially expressed genes (DEGs) in integrated bulk profiles of COAD patients from the Gene Expression Omnibus (GEO) database (GSE18462, GSE23878, and GSE39084 datasets) via integrative bioinformatics analysis. Next, we used a deep-learning algorithm involving self-organizing maps (SOMs) facilitate identification of molecular subgroups based on the SN-associated DEGs. Then, from a set of 101 machine-learning algorithm combinations, we identified random survival forest (RSF) and ridge regression as the optimal pipeline for constructing the SN-associated predictive model from The Cancer Genome Atlas colon adenocarcinoma (TCGA-COAD) cohort and validating it in a bulk profile from the GEO (GSE146771 dataset). Furthermore, we used cox regression to identify NOX4 as the SN-related hub gene involved in COAD pathogenesis. The molecular and immune features of NOX4 were also deciphered at the bulk and single-cell levels in COAD and in AI-powered virtual cells. The AI-driven multilayer network facilitated recognition of talazoparib that targets NOX4 for the treatment of COAD. Finally, the *in vitro* assays validated the pathogenic role of NOX4-based COAD for regulating the neutrophil-malignant cell axis. Thus, our study offers novel insights into the integration of succinylation and neutrophilic causes to forecast the overall survival (OS) of COAD patients and enrich the therapeutic approaches, thereby providing opportunities for clinical translation in COAD. The workflow of this study is depicted in Figure 1.

## 2 Materials and methods

### 2.1 Bulk profile sources

Colorectal tumor tissue data of patients with COAD containing paired bulk profiles of para-cancer tissues (GSE18462, GSE23878, and GSE39084 datasets) were downloaded from the GEO database via the GEOquery package of R (Davis and Meltzer, 2007). All three datasets were generated on the GPL570 platform. We then integrated these datasets and performed normalization using the ComBat function from the sva and limma packages of R (Leek et al., 2012; Ritchie et al., 2015). After integration, we identified 26 normal samples and 107 COAD patient samples in the combined dataset. We designated this integrated bulk profile for acquiring the SN-associated shared DEGs. In addition, we obtained the STAR-counts data and relevant clinical information for COAD tumors from TCGA-COAD database. Subsequently, we extracted the data in the transcripts per million (TPM) format and performed normalization through transformation via the limma package (Ritchie et al., 2015). The colorectal tumor tissue bulk profiles of the COAD patients (GSE17536 and GSE17537 datasets) were also integrated using the sva and limma packages, which resulted in 232 COAD patient samples (Ritchie et al., 2015). We designated TCGA-COAD was used as the training set, and the integrated GSE17536 and GSE17537 datasets were used as the independent validation set. The succinylation-associated gene list was acquired from the GeneCards database by setting the threshold to greater than 1. Moreover, immunohistochemistry (IHC) samples of COAD patients and normal controls were acquired from the human protein atlas (HPA) database.

### 2.2 Identification of succinylation-related DEGs

Following the preprocessing stage, which was visualized using the box and principal component analysis (PCA) plots, the DEGs were identified from the combined GSE18462, GSE23878, and GSE39084 datasets using the limma package by setting *p*-value <0.05 and log of fold change (FC) >0.5 (Ritchie et al., 2015; Zhou et al., 2026). The findings were observed with a volcano plot created using the ggplot2 package in R (Zhou et al., 2026). The DEGs were then intersected with the succinylation-related gene list to identify the succinylation-associated DEGs. Additionally, KEGG and GO enrichment analyses were conducted to assess the molecular functions of the these identified DEGs by employing the hallmark gene set obtained from the MsigDB database through the clusterProfiler package in R software (Yu et al., 2012).

### 2.3 Identification of SN-related shared DEGs

Single-sample gene-set enrichment analysis (ssGSEA) was performed in R with an outstanding algorithm to determine the infiltration levels of various immune cell types within a cellular mixture using transcriptomic data as the foundation. Here, we first examined the proportions of 22 distinct immune cell types in both normal and COAD samples derived from the integrated bulk dataset (Zhou et al., 2023). Subsequently, we performed weighted gene coexpression network analysis (WGCNA) to discern the modules containing highly correlated genes, clarify the relationships among these modules, and elucidate their associations with external sample characteristics (Langfelder and Horvath, 2008). This approach ultimately aims to identify the potential target genes associated with the phenotype. In our analysis, WGCNA was implemented using the WGCNA package in R to pinpoint modules displaying the strongest correlations with neutrophils in patients diagnosed with COAD (Langfelder and Horvath, 2008). Initially, we preprocessed the sample data to exclude outliers; then, we generated a correlation matrix using the WGCNA package (Langfelder and Horvath, 2008). The ideal soft threshold was determined to convert the correlation matrix into an adjacency matrix, which subsequently facilitated the creation of a topological overlap matrix (TOM) (Langfelder and Horvath, 2008). Next, a phase dissimilarity metric based on the TOM was employed to group the genes exhibiting analogous expression patterns into distinct gene modules via average linkage hierarchical clustering (Langfelder and Horvath, 2008). This showed that one module exhibited the strongest correlation with neutrophils (Langfelder and Horvath, 2008). Ultimately, we intersected the succinylation-associated DEGs with the shared module of neutrophils to identify the SN-related shared DEGs. We performed friend and genetic variation analyses to identify the gene importance and genetic landscape (Xu et al., 2023; Xu et al., 2025). Lastly, the MCODE plugin of Cytoscape was used to select the SN-associated hub DEGs (Wu et al., 2024).

### 2.4 Machine-learning algorithm for constructing predictive model

We designated TCGA-COAD cohort data as the training set and the combination of GSE17536 and GSE17537 datasets as the independent validation set. We then developed a prognostic model based on TCGA-COAD cohort and validated it using the combined GSE17536 and GSE17537 datasets by employing a machine-learning-based analytical framework incorporating 10 distinct methods and 101 model combinations; in these evaluations, we utilized both leave-one-out cross-validation (LOOCV) and 10-fold cross-validation strategies to enhance reliability (Wang et al., 2024; Zhong et al., 2025). For each model configuration, the concordance index (C-index) was calculated using TCGA-COAD dataset in conjunction with the GSE17536 and GSE17537 datasets (Wang et al., 2024). The configuration demonstrating the highest C-index value across the two profiles was deemed as the most optimal (Wang et al., 2024). Upon confirming the ideal model, we conducted Kaplan–Meier (KM) curve analysis, receiver operating characteristics (ROC) curve analysis, risk factor analysis, and calibration analysis to evaluate the predictive capability of the model for OS of the patients using the survival, survminer, and timeROC packages within R (Wang et al., 2024). Multivariate analysis was performed for selecting prognostic indicators among the SN-associated hub DEGs (Wang et al., 2024). Using TCGA-COAD cohort along with the expression and methylation of the prognostic indicators and their associations with clinical outcomes, we assessed the immune and stromal infiltration via the GEPIA2 and GSCA databases alongside the CIBERSORT and ESTIMATE algorithms in R (Wang et al., 2024; Li et al., 2025).

### 2.5 SOM-based deep-learning framework for identifying risk subgroups

The SOM is an artificial-neural-network-based deep-learning algorithm used for character classification and was implemented through the kohonen package in R (Licen et al., 2023). We acquired the STAR-counts data along with relevant clinical information pertaining to COAD tumors from TCGA database. Subsequently, we transformed the data into TPM format and performed normalization via the log_2_ (TPM + 1) transformation, in accordance with the methodology outlined previously. Using TCGA-COAD cohort as the foundational dataset, we applied SOM to discern the novel molecular subgroups following established protocols (Licen et al., 2023). After identifying the diverse molecular subgroups within TCGA-COAD cohort, we analyzed the KM plots, clinical significance, tumor stemness, tumor purity, and checkpoint expression for the identified subgroups (Wang et al., 2024; Zeng et al., 2025a).

### 2.6 Single-cell transcriptomic analysis

We acquired the single-cell transcriptomic dataset related to COAD (GSE146771) from the GEO repository. The analysis of the scRNA-seq data involved several essential phases, including quality control (QC), dimensionality reduction, and marker identification, all of which were performed using the Seurat package in R (Butler et al., 2018; Ding et al., 2024). The QC process was rigorously applied to each cell by adhering to predetermined criteria as follows: the gene counts were restricted to a range between 200 and 6,000; the unique molecular identifier (UMI) counts were mandated to exceed 1,000; the proportion of mitochondrial gene expression was maintained below 10% (Butler et al., 2018). Following the QC procedures, the data were normalized, which enabled the identification of 2,000 genes exhibiting significant variability for further analysis (Butler et al., 2018). The dimensionality reduction techniques particularly included t-distributed stochastic neighbor embedding (t-SNE) and uniform manifold approximation and projection (UMAP) (Butler et al., 2018). The cell type annotations were performed using the scMayoMap algorithm within R (Yang et al., 2023). The expression levels of the target genes were assessed across various annotated cell populations. The copy number variation (CNV) within individual cells was precisely evaluated using the inferCNV package in R, with particular focus on epithelial cells to discern the cancerous cells with reference to T cells (Zhou et al., 2026). The communication patterns among various cell types were inferred using the CellChat package in R (Jin et al., 2021). Moreover, we explored the energy metabolic pathways among the cell populations at the single-cell level by employing the SCENITH package in R (Argüello et al., 2020). Additionally, ssGSEA was conducted to investigate the functional enrichment of hub genes at single-cell resolution using the hallmark gene set obtained from the MsigDB database via the clusterProfiler package (Yu et al., 2012). Lastly, pseudo-time analysis of the target gene expression within specific cell types was performed using the Monocle2 package in R (Huang et al., 2023). The upstream and downstream gene lists of the target genes in specific cells were analyzed using the scTenifoldKnk package in R (Osorio et al., 2022). Additionally, KEGG and GO enrichment analyses were performed to estimate the molecular and biological functions associated with these genes via the clusterProfiler package (Yu et al., 2012).

### 2.7 AI-guided drug design and repurposing pipeline

The SN-linked hub genes were prioritized using an AI-guided composite target evidence score integrating prognostic association, neutrophil/immune coupling, and network hubness (along with single-cell support and *in silico* perturbation impact where available) (Seifert et al., 2023). The components were scaled to the range of 0–1 and aggregated as a weighted sum (Seifert et al., 2023). A NOX4-centered multilayer network was assembled from curated interaction evidence together with SN-associated coexpression links and decomposed into functional modules by community detection, with quantification of the module composition (up/down/neutral in SN high-risk), pathway enrichment (false discovery rate (FDR)-controlled), and druggability density (Seifert et al., 2023). Repurposing candidates were then ranked using an integrated drug priority score combining NOX4 network linkage, predicted reversal of SN-risk transcriptional programs, and model-based response estimation, with all components being normalized to the range of 0–1 (Seifert et al., 2023). The top candidates underwent *in silico* ADMET/PK and toxicity profiling, and a developability score was computed as the fraction of endpoints meeting favorable criteria (NA endpoints excluded) scaled to 0–1 using a predefined pass threshold (e.g., ≥0.60) (Seifert et al., 2023). Finally, structure-based evaluation was performed by docking talazoparib and comparator candidates to the NOX4 structural model (source specified) and by reporting the representative poses, interaction fingerprints, and comparative docking score distributions versus the top candidates and decoys; experimental validations were not performed in this study (Seifert et al., 2023).

### 2.8 Cell lines and culture conditions

HL-60, SW480, and HEK293T cell lines were purchased from Biointron (China). The HL-60 cell line can be effectively induced to differentiate into neutrophils using 1 μM all-trans retinoic acid (ATRA) for 5–7 d. The HL-60 cells were cultured in RPMI 1640 complete medium supplemented with a 1% antibiotic mixture of penicillin-streptomycin and 10% fetal bovine serum (FBS, Gibco), before collecting the supernatant for further analysis. The SW480 cells were cultured in RPMI 1640 complete medium supplemented with a 1% antibiotic mixture of penicillin-streptomycin and 10% FBS. To simulate the neutrophil vs. cancer cell microenvironment, we collected 3 mL of the supernatant of HL-60 and added it to the SW480 cells. The HEK293T cell line was maintained in Dulbecco’s modified Eagle medium (DMEM) enriched with 1% penicillin-streptomycin mixture and 10% FBS. All cell-passage procedures were conducted under controlled conditions of 37 °C and 5% CO_2_ within a humidified incubator.

### 2.9 qRT-PCR

Total RNA isolation was performed with TRIzol reagent (TaKaRa, Beijing, China), followed by quantification and quality assessment of the RNA concentration, purity, and integrity. Subsequently, 1 µg of the total RNA was used for reverse transcription using HiScript II Q RT SuperMix for qPCR (+gDNA wiper) in combination with a gDNA eraser (Vazyme, Shanghai, China). The resulting cDNA sample was evaluated for concentration, purity, and integrity. Then, quantitative real-time PCR was conducted using the SYBR Green MasterMix (catalog no. 11203ES50, Yeasen, Shanghai, China) through 40 amplification cycles (Zeng et al., 2025b). The gene expression was quantified using the ΔΔCt method, with GAPDH serving as the internal reference. The specific primer sequences employed in the qRT-PCR analysis are listed in Table 1.

**TABLE 1:** Primers used in this study.

Gene name	Primer (5′-3′)
*NOX4*	F: GAAGACAGATGAGCTTCCCCA R: GGTTGGTGATGAAGGGGGTC
*FAS*	F: GGCAAAAGAAGAAGACAAAGCC R: CCTGTACGCCAACACAGTGC
*ICAM1*	F: CCTGTTTCCTGCCTCTGAAG R: GGGTTCTGTCGAACTCCTCA
*CXCL10*	F: CACCTCCAGTCTCAGCACCATGA R: TGCAGGTACAGCGTACAGTTCT
*IL-8*	F: AACTTCTCCACAACCCTCTG R: TTGGCAGCCTTCCTGATTTC
*CCL5*	F: GCATCCCTCACCGTCATCCTC R: GCACTTGCTGCTGGTGTAAAA
*GAPDH*	F: GAGAAGGCTGGGGCTCATTT R: ATGACGAACATGGGGGCATC

### 2.10 Silencing via shRNA

The shRNA oligonucleotides were inserted into the pLKO.1 lentiviral backbone. In brief, the HEK293T cells were co-transfected with the shRNA-containing plasmids along with the VSV-G envelope and psPAX2 packaging plasmids by employing Lipofectamine 2000 (Thermo Fisher Scientific) according to manufacturer instructions. After 24 h, the transfection medium was refreshed. The viral particles were collected 72 h later, passed through a 0.45-μm filter, and subsequently utilized for target cell infection with 4 μg/mL polybrene (Sigma-Aldrich) supplementation. In the lentiviral infection procedure, differentiated HL-60 cells were seeded in 24-well plates at 5 × 10^4^ cells per well and allowed to grow until achieving 50%–70% coverage. The culture medium was exchanged with sh-NOX4 lentiviral preparations. Following a 72-h exposure period, the infected cells were detached using trypsin, rinsed with phosphate-buffered saline (PBS), and transferred to 10-cm dishes at a seeding density of 500 cells each. Puromycin (Thermo Scientific) was used in each culture dish as a selective agent over a 3-week period to remove cells that failed to undergo successful transduction. Monoclonal populations that persisted were recognized through the appearance of distinct circular growth patterns; these were cultured further and subjected to serial dilution cloning to generate permanent cell lines with reduced target gene expression. The shRNA sequence is as follows:

ShRNA1: CCT​GTG​GTG​TTA​CTA​TCT​GTA.

### 2.11 Western blotting

Following the experimental procedures, the cellular samples were thoroughly rinsed with chilled PBS (HyClone, Seattle, Washington, United States) before being harvested through careful scraping. Total protein isolation was then performed by employing RIPA lysis solution (Beyotime, Shanghai, China) supplemented with both phosphatase and protease inhibitors (Beyotime). The resulting cellular suspension was subjected to centrifugation at 14,000 × *g* for 15 min under 4 °C conditions. The obtained supernatant was then combined with 5× SDS-PAGE loading buffer (Beyotime) and heat-treated in boiling water for 10 min to achieve protein denaturation. Electrophoretic separation was conducted using SDS-PAGE, followed by protein transfer onto a polyvinylidene fluoride membrane (Beyotime) for subsequent immunoblotting. Prior to antibody incubation, the membrane was treated with a blocking solution (NcmBlot, NCM Biotech, Suzhou, China) for 10 min; the primary antibody prepared in a 5% solution of bovine serum albumin was then applied to the membrane and allowed to bind overnight at 4 °C. The membrane was subsequently treated with a secondary antibody solution (Thermo Fisher Scientific, Waltham, Massachusetts, United States) for 2 h at ambient temperature; this solution was prepared by diluting the antibody in the ratio of 1:1,000 in Western blot secondary antibody dilution buffer (Beyotime). The target proteins were visualized through enhanced chemiluminescence (ECL) detection reagents (Thermo Fisher Scientific). The protein expression levels were quantitatively assessed by measuring the band intensity with ImageJ software (version 1.57), with GAPDH serving as the loading control. The following primary antibodies were employed in the analysis: NOX4 (catalog no. ab289740, Abcam, United States, 1:2,000 dilution) and β-actin (catalog no. ab6276, Abcam, 1:5,000 dilution). All reagents were obtained from Solarbio (Beijing, China).

### 2.12 Flow cytometry

Approximately 1 × 10^6^ cells were suspended in PBS and stained with 5 μL of mouse anti-human Arg1 monoclonal antibodies (catalog no. ab212522, ABCAM, 1:1,000 dilution) for 30 min. Next, PE-labeled secondary antibodies (catalog no. MA1-10377, Thermo Fisher Scientific) were added, and the mixture was incubated for 20 min. After washing, the cells were resuspended in PBS and analyzed using a CytoFLEX flow cytometer (Beckman, Brea, California, United States).

### 2.13 Cell counting kit-8 (CCK-8) assays

Talazoparib was purchased from MCE company (China). Cells in the logarithmic phase of growth were subjected to trypsinization, followed by enumeration and subsequent seeding onto 96-well plates at a concentration of 3,000 cells per well (n = 6). Following incubation at predetermined intervals (4, 24, 48, 72, 96, and 120 h), 10 µL of CCK-8 reagent (catalog no. C0038, Beyotime) was introduced to each well, and the plates were incubated for an additional 2 h. The absorbance readings were obtained at 450 nm using a microplate reader (EnSight, PerkinElmer, United States). The cell proliferation rate was determined using formula [(A_d − A_blank_d) / (A_4 h − A_blank_4 h)]. All experiments were conducted in triplicate to ensure reliability of the results. For IC_50_ validation, the cells were seeded in 96-well plates at a density of 2,000 cells per well (100 µL per well) and cultured for 24 h (at 37 °C and 5% CO_2_) following drug treatment. Subsequently, 10 µL of the CCK-8 reagent was added to each well while taking care to avoid bubble formation. The plates were then returned to the incubator for 2 h. The absorbance values were measured at 450 nm using a microplate reader (EnSight, PerkinElmer, United States), and the cell survival rates were calculated according to manufacturer protocol.

### 2.14 Statistical analysis

In the bioinformatics analysis, all statistical assessments were performed using R software (version 4.2.2). Pearson correlation analysis was used to investigate associations among various variables by defining the statistical significance as *p*-value or FDR below 0.05. The data are expressed as mean ± standard deviation (SD), with the significance levels being indicated as *p < 0.05, **p < 0.01, and ***p < 0.001. For the experimental components, all statistical analyses were carried out using GraphPad Prism (version 8.0.2). Each experiment was conducted with at least three biological replicates, and the results are reported as mean ± SD. Differences between two datasets were assessed using either two-way ANOVA or Student’s t-test, with a *p*-value less than 0.05 being considered statistically significant.

## 3 Results

### 3.1 Identification of succinylation-related DEGs in COAD patients

**FIGURE 2:**
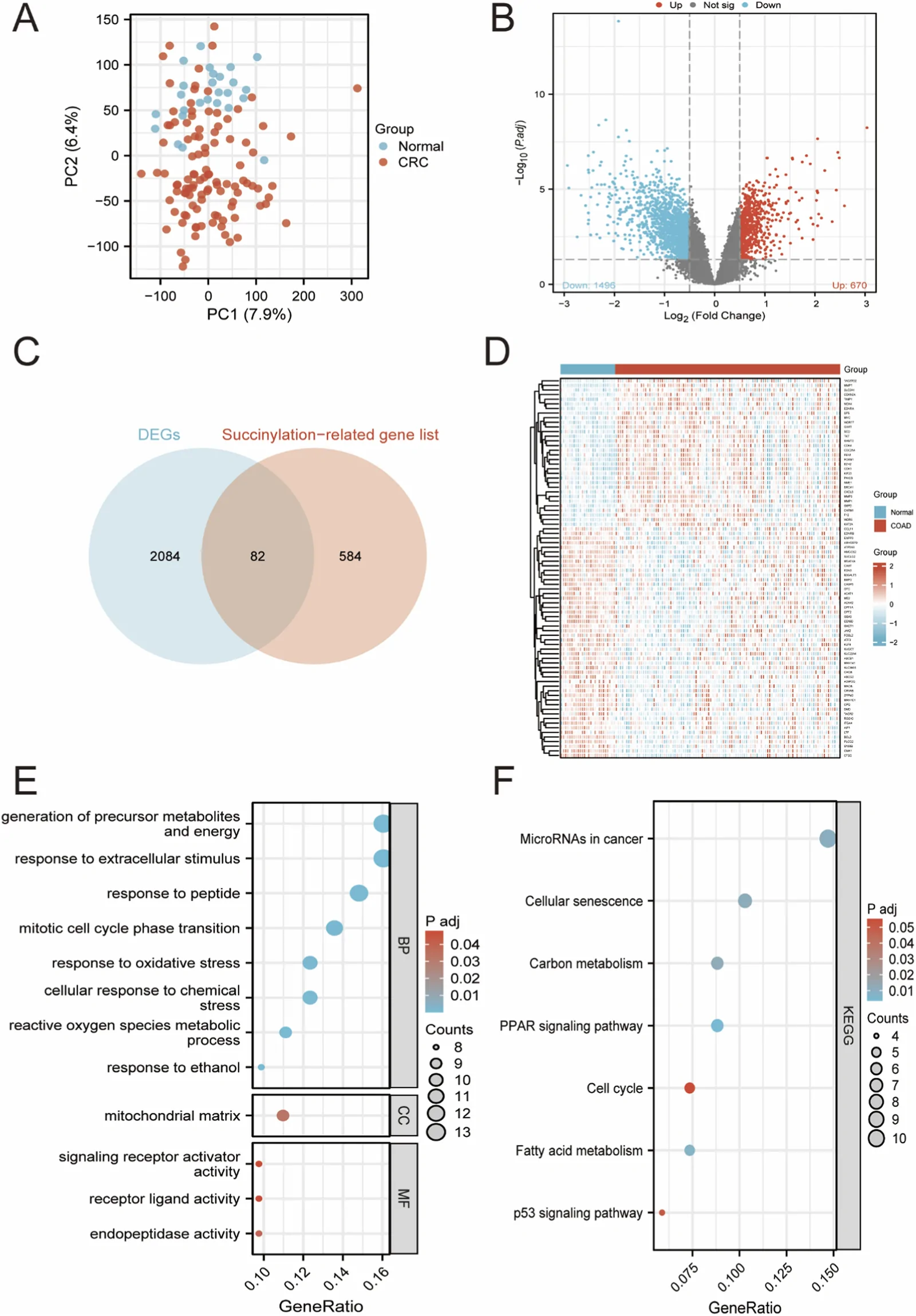
Identification of succinylation-associated DEGs. **(A)** Principal component analysis (PCA) plot of the GSE10072 dataset. **(B)** Upregulated and downregulated differentially expressed genes (DEGs) in the integrated dataset. **(C)** Identification of succinylation-associated DEGs. **(D)** Expression patterns of the succinylation-associated DEGs. **(E–F)** GO and KEGG enrichment analyses of the succinylation-associated DEGs.

After integrating the GSE18462, GSE23878, and GSE39084 datasets, we identified 670 upregulated and 1,496 downregulated DEGs (Figures 2A,B). Next, the succinylation-related gene list was intersected with the DEGs to identify 82 succinylation-associated DEGs (Figure 2C), and these were evaluated for their expression patterns along with the corresponding molecular and biological functions (Figures 2D–F).

### 3.2 Identification of SN-associated DEGs in COAD patients

**FIGURE 3:**
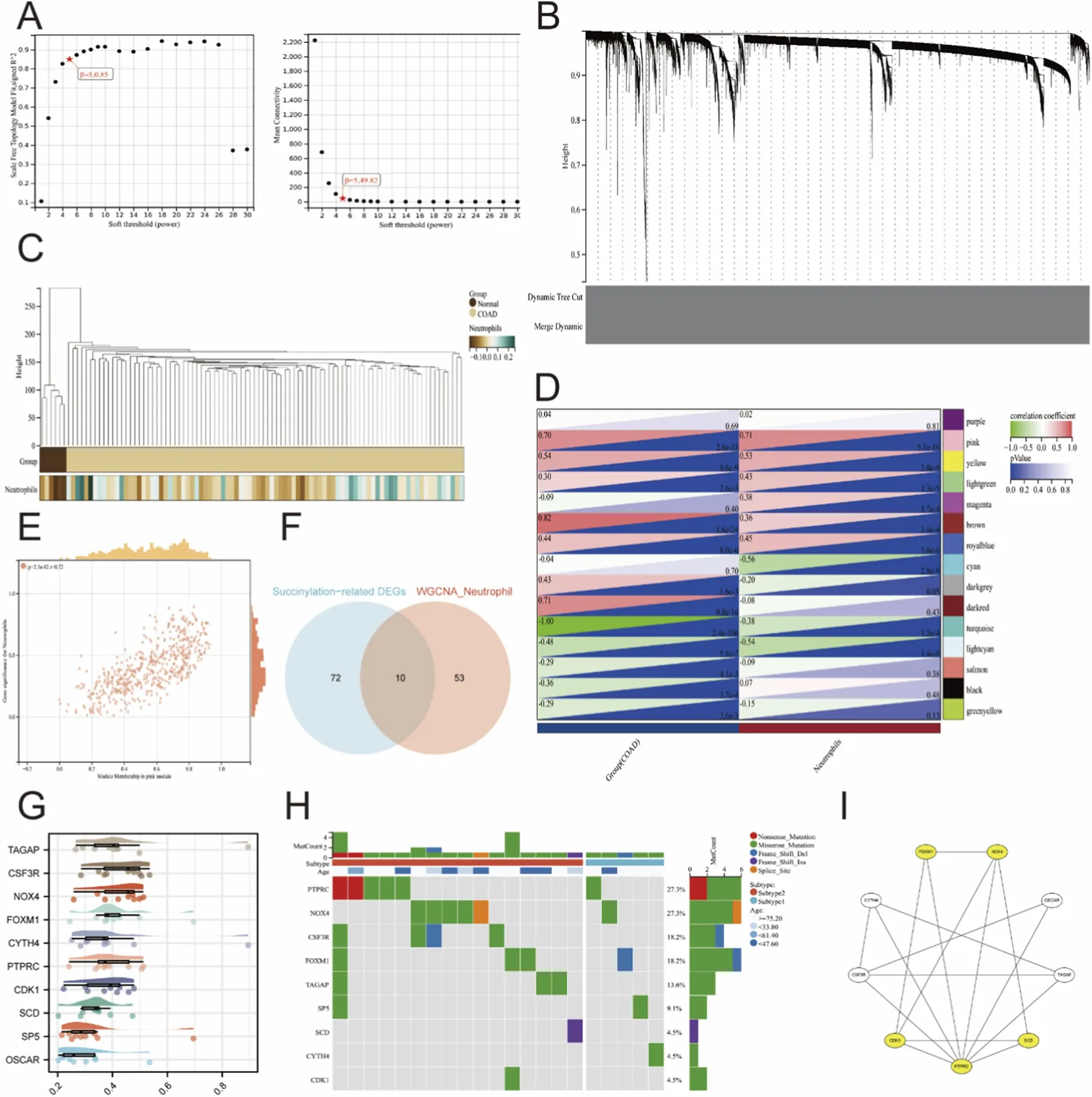
Identification of succinylation–neutrophil (SN)-associated shared DEGs in colorectal carcinoma (COAD) patients. **(A)** Scatterplot of the representative modules in the integrated dataset. **(B)** Gene coexpression modules shown with different colors under the gene tree. **(C)** Intersection between the succinylation-related gene list and DEGs from the integrated dataset. **(D,E)** Module clustering tree and trait relationship heatmap generated by weighted gene coexpression network analysis (WGCNA) of the integrated dataset. **(F)** Identification of SN-associated shared DEGs. **(G)** Friend analysis. **(H)** Genetic variation analysis. **(I)** Protein–protein interaction (PPI) network of the shared DEGs.

By employing the ssGSEA immune infiltration algorithm in combination with the integrated bulk profile, we constructed an unstructured coexpression network using WGCNA to identify modules demonstrating the strongest associations with neutrophils in COAD. To ascertain the most suitable “soft” threshold β, we determined the values (5, 0.85) and (5, 49.82) for scale independence and 0.87 for average connectivity (Figure 3A). Subsequently, we generated a clustering dendrogram for both the disease and control groups (Figures 3B,C). By setting the module merging threshold as 0.25 and minimum module size as 60, we successfully delineated 15 distinct gene coexpression modules, each of which is represented by a unique color in Figure 3D. Importantly, we observed that the pink module exhibited significant correlations with neutrophils (Figure 3E). Additionally, we performed an intersection of the DEGs associated with the highly correlated modules, which resulted in the identification of 10 shared DEGs (Figure 3F). Next, we estimated the importance and genetic variation of these shared DEGs (Figures 3G,H). Finally, we constructed a protein–protein interaction (PPI) network and identified five SN-associated DEGs (Figure 3I).

### 3.3 Construction of SN-associated prognostic model for COAD patients

**FIGURE 4:**
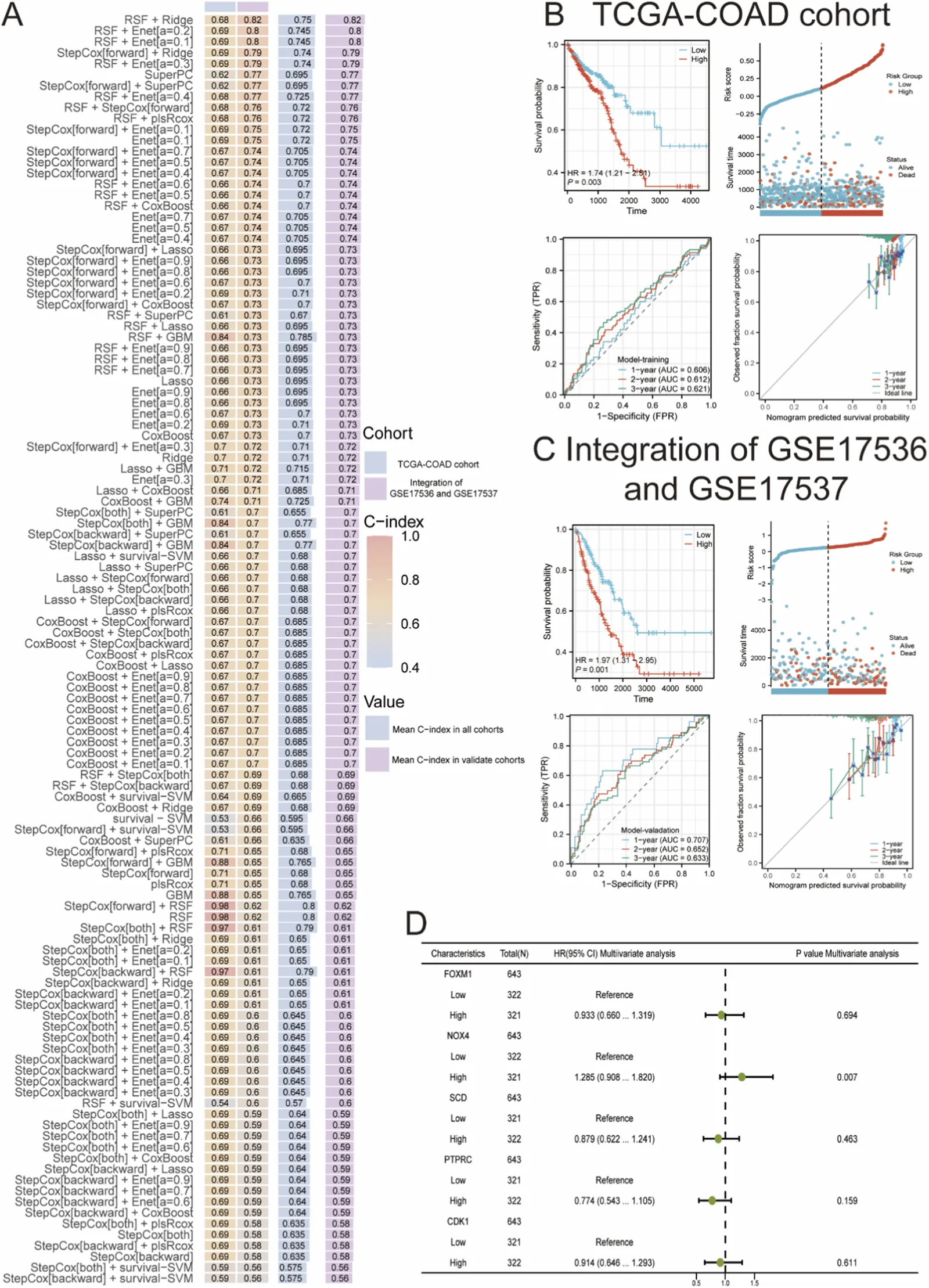
Construction of SN-associated prognostic model for COAD patients. **(A)** Tenfold cross-validation framework enables concordance index (C-index) calculations for the 101 distinct combinations of prediction models. **(B)** Overall survival in the high-risk and low-risk groups in TCGA-COAD cohort. **(C)** Overall survival in the high-risk and low-risk groups in the integrated bulk profile. **(D)** Multivariate cox regression analysis.

Using TCGA-COAD dataset as the training cohort, we constructed a total of 101 models through LOOCV. Our analysis revealed that the integration of RSF and ridge regression techniques yielded the most efficient model and attained the highest concordance index (C-index = 0.82), as depicted in Figure 4A. This model was designated as the SN-associated risk predictive model and was used to effectively stratify patients into high-risk and low-risk categories within both TCGA-COAD and combined GSE17536 and GSE17537 cohorts. Further assessments, including survival and ROC analyses, indicated a significant correlation between the SN-associated risk score and adverse clinical outcomes, as illustrated in Figures 4B,C. Moreover, calibration analysis illustrated that this risk-stratified model possessed satisfactory accuracy (Figures 4B,C). Multivariate cox regression analysis confirmed that NOX4 can be considered as a central prognostic indicator involved in COAD pathogenesis (Figure 4D).

### 3.4 Identification of SN-associated risk groups in COAD patients

**FIGURE 5:**
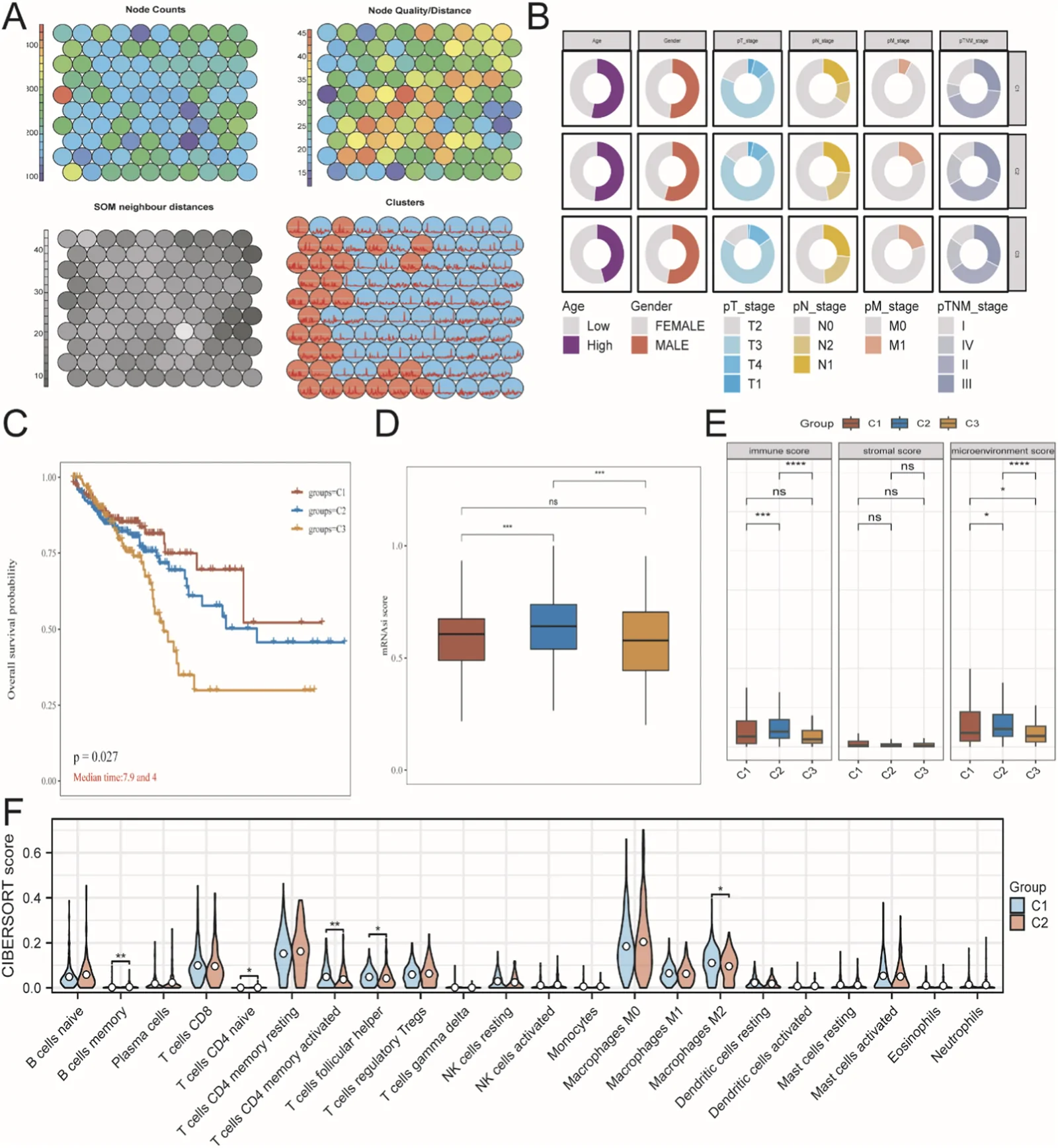
**(A)** Results of the self-organizing map (SOM) deep-learning clustering algorithm. **(B)** Confirmation of clinical conditions among the subgroups. **(C)** Kaplan–Meier analysis. **(D)** Tumor stemness analysis. **(E)** Immune and stromal infiltration analysis. **(F)** Immune checkpoint expression analysis.

To enhance the stratification under COAD, we applied deep-learning SOMs to TCGA-COAD cohort to identify distinct risk groups based on the SN-associated hub genes (Figure 5A). Thus, we discovered three novel molecular groups, namely, C1, C2, and C3, where C3 illustrated the worst prognosis compared to C1 and C2 (Figure 5C). We next identified patients in these three distinct groups and compared their tumor stemness, immune infiltration, and checkpoint expression along with the corresponding clinical parameters (Figures 5B,D–F).

### 3.5 Landscape of COAD tumor heterogeneity at the single-cell level

**FIGURE 6:**
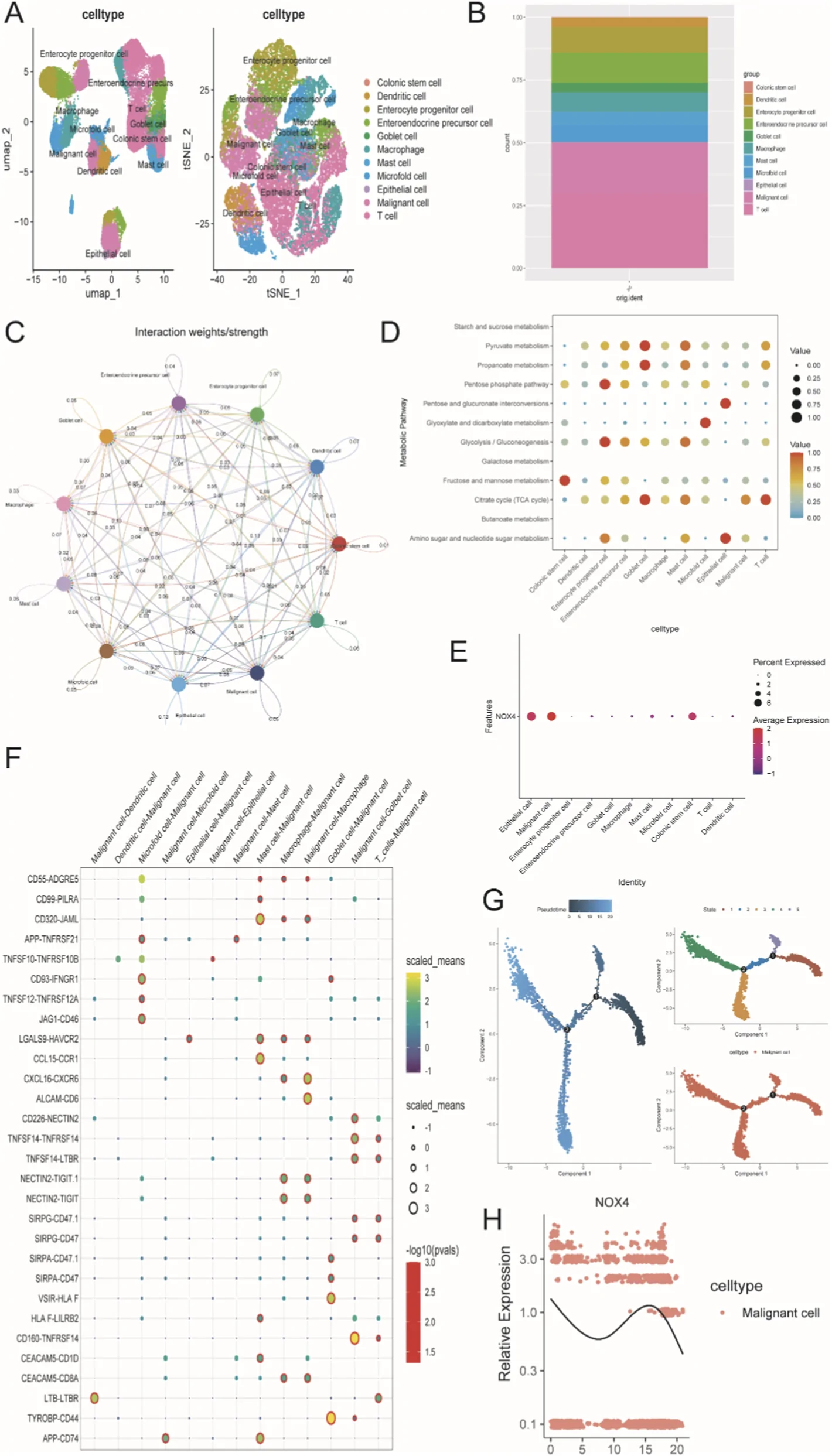
Landscape of COAD tumor heterogeneity at the single-cell transcriptomic level in COAD patients. **(A)** Cell annotation analysis. **(B)** Cell proportion analysis. **(C)** CellChat patterns among the identified cell types. **(D)** Energy metabolism analysis. **(E)** Distribution of NOX4 in various cell types. **(F)** Ligand–receptor interactions between malignant cells and other cell types. **(G,H)** Monocle2 analysis of the malignant cells and NOX4 in malignant cells.

The scRNA-seq data utilized in this investigation were sourced from the GS146771 dataset. Initially, the data were normalized and subjected to QC procedures (Supplementary Figures S1A–D). To enhance characterization of the cell types, we categorized the cells into 25 distinct clusters based on the expression of established markers (Supplementary Figures S1E–G). Following annotation, we confirmed the presence of 11 unique cell types (Figure 6A). Furthermore, T cells and malignant cells accounted for the largest proportions among the cell types (Figure 6B). We estimated the energy metabolism patterns and cell chat patterns among these 11 cell types (Figures 6C,D). It is seen that NOX4 was mainly distributed in the malignant cells (Figure 6E); hence, we examined the molecular patterns and interactions between malignant cells and other cell types (Figure 6F). Lastly, the pseudo-time trajectories (particularly for malignant cells and NOX4 expression during malignant cell differentiation) were estimated (Figures 6G,H).

### 3.6 NOX4 molecular and immune features in COAD patients

**FIGURE 7:**
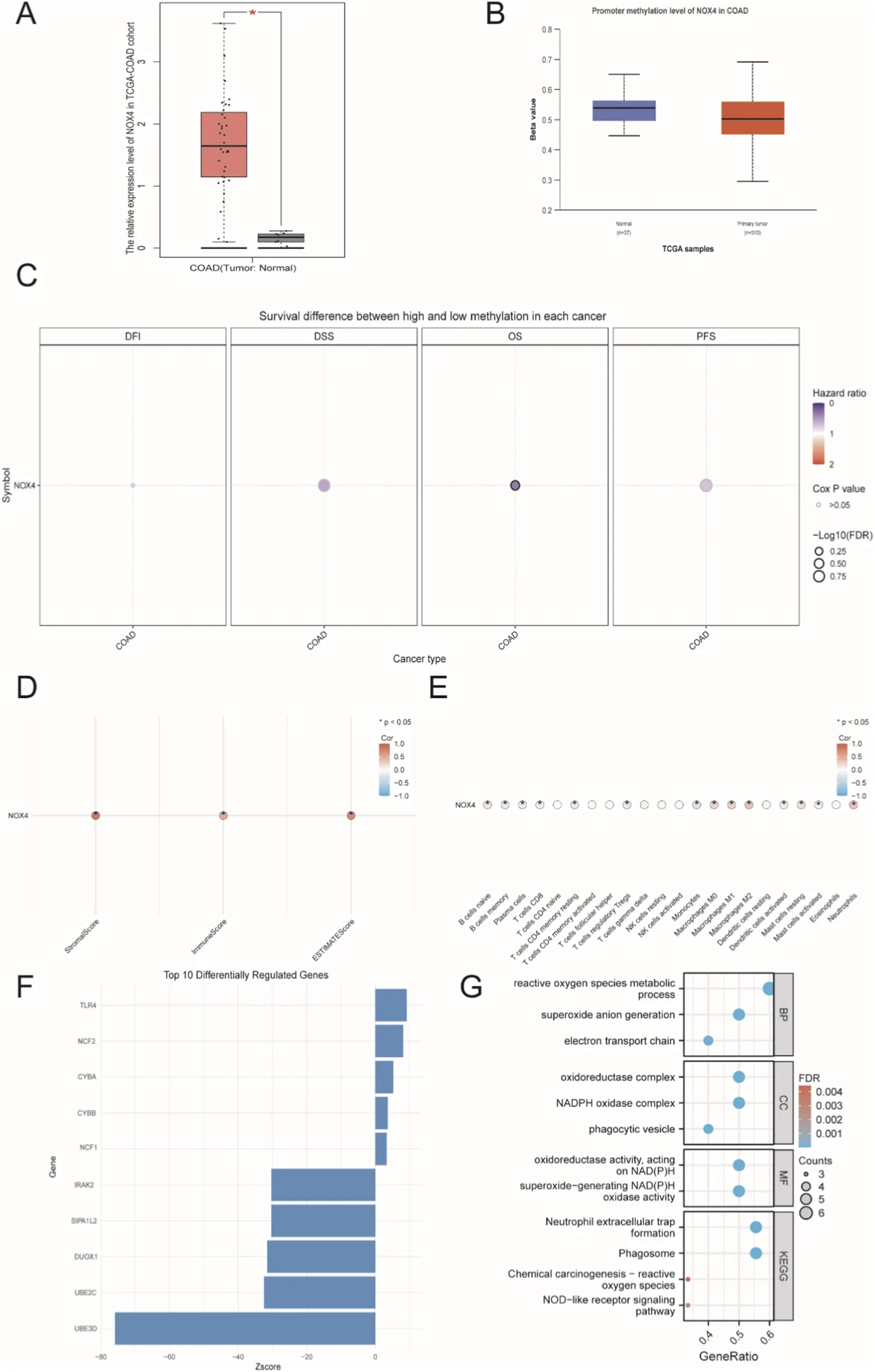
NOX4 molecular and immune landscapes in COAD patients. **(A)** *NOX4* expression in COAD patients. **(B)** Methylation level of NOX4 and **(C)** associations between methylation levels and clinical outcomes in COAD. Association of NOX4 with **(D)** immune and **(E)** stromal infiltration. **(F)** Knockdown of NOX4 in malignant cells. **(G)** Single-cell gene-set enrichment analysis of NOX4.

We first explored the NOX4 expression levels in COAD tumor patients compared to the normal group (Figure 7A) and found lower methylation levels of NOX4 in the COAD group along with worse clinical outcomes (Figures 7B,C). We also estimated the associations of NOX4 with immune and stromal proportions (Figures 7D,E). Then, we performed AI-driven knockout of NOX4 in the malignant cells and identified the top-10 genes with expression changes, along with the corresponding interaction and functional patterns (Figure 7F). Moreover, we performed single-cell GSEA of NOX4 to gain insights into the molecular and biological functions among the 11 cell types (Figure 7G).

### 3.7 AI-driven drug repurposing framework for colorectal cancer: NOX4 target nomination and talazoparib evaluation

**FIGURE 8:**
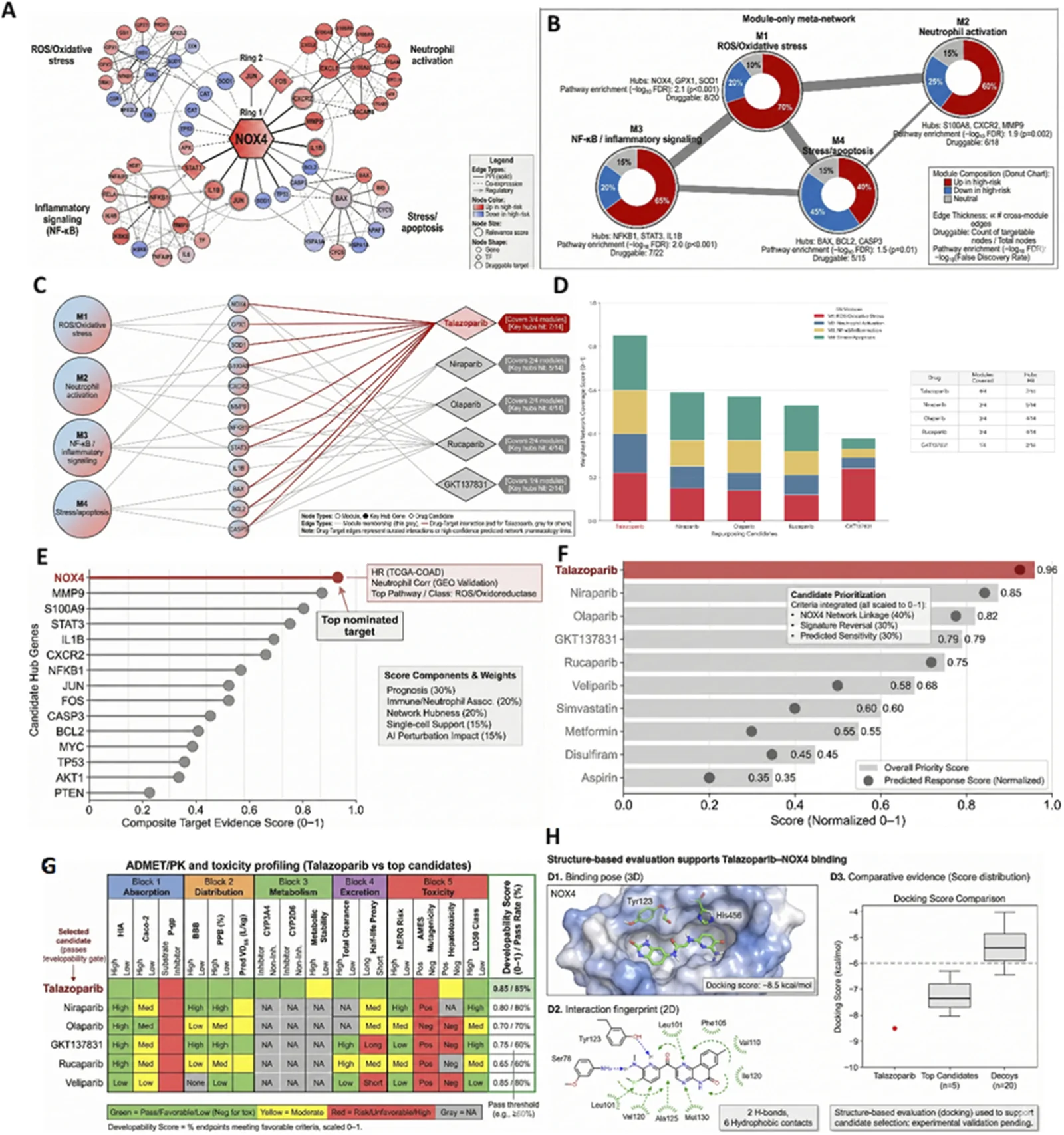
Artificial intelligence (AI)-guided network-to-candidate drug repurposing for colorectal cancer identifies NOX4 and prioritizes talazoparib. **(A)** Multilayer NOX4-centered SN network highlights reactive oxygen species (ROS), neutrophil activation, NF-κB signaling, and stress/apoptosis modules. **(B)** Module meta-network summarizes inter-module connectivity, SN-risk directionality, pathway enrichment (−log_10_ (FDR)), and druggability density. **(C)** Drug–target–module overlay shows the broad cross-module coverage and key-hub engagement of talazoparib versus comparator candidates. **(D)** Weighted multitarget coverage index quantifies the module breadth and hub impact across candidates. **(E)** AI target nomination ranks SN-linked hubs by composite target evidence score with NOX4 prioritization. **(F)** Integrated candidate ranking (NOX4 linkage, signature reversal, and predicted response) selects talazoparib as the top candidate. **(G)** ADMET/PK–toxicity heatmap and developability score support the advancement of talazoparib. **(H)** Structure-based evaluation shows a plausible talazoparib–NOX4 pose, interaction fingerprint, and favorable docking distribution (validation pending).

To align SN-risk biology with drug-design decision-making, we integrated multilayer network evidence, AI-based target nomination, and candidate evaluation into a single network-to-candidate framework (Figure 8). The NOX4-centered SN network revealed modular crosstalk across the ROS/oxidative stress, neutrophil activation, NF-κB inflammation, and stress/apoptosis programs (Figures 8A,B), motivating a multitarget coverage perspective for repurposing. The drug–target–module overlays and weighted multitarget coverage index were used to quantify cross-module reach and hub engagement among the drug candidates (Figures 8C,D), while AI target nomination prioritized NOX4 among the SN-linked hubs (Figure 8E). By integrating NOX4 linkage, signature reversal, and predicted response, we identified talazoparib as the top repurposing candidate (Figure 8F), which was further supported by the ADMET/toxicity profiling and structure-based evaluation (Figures 8G,H).

### 3.8 *In vitro* assays to identify NOX4 association with N2-like neutrophil activation

**FIGURE 9:**
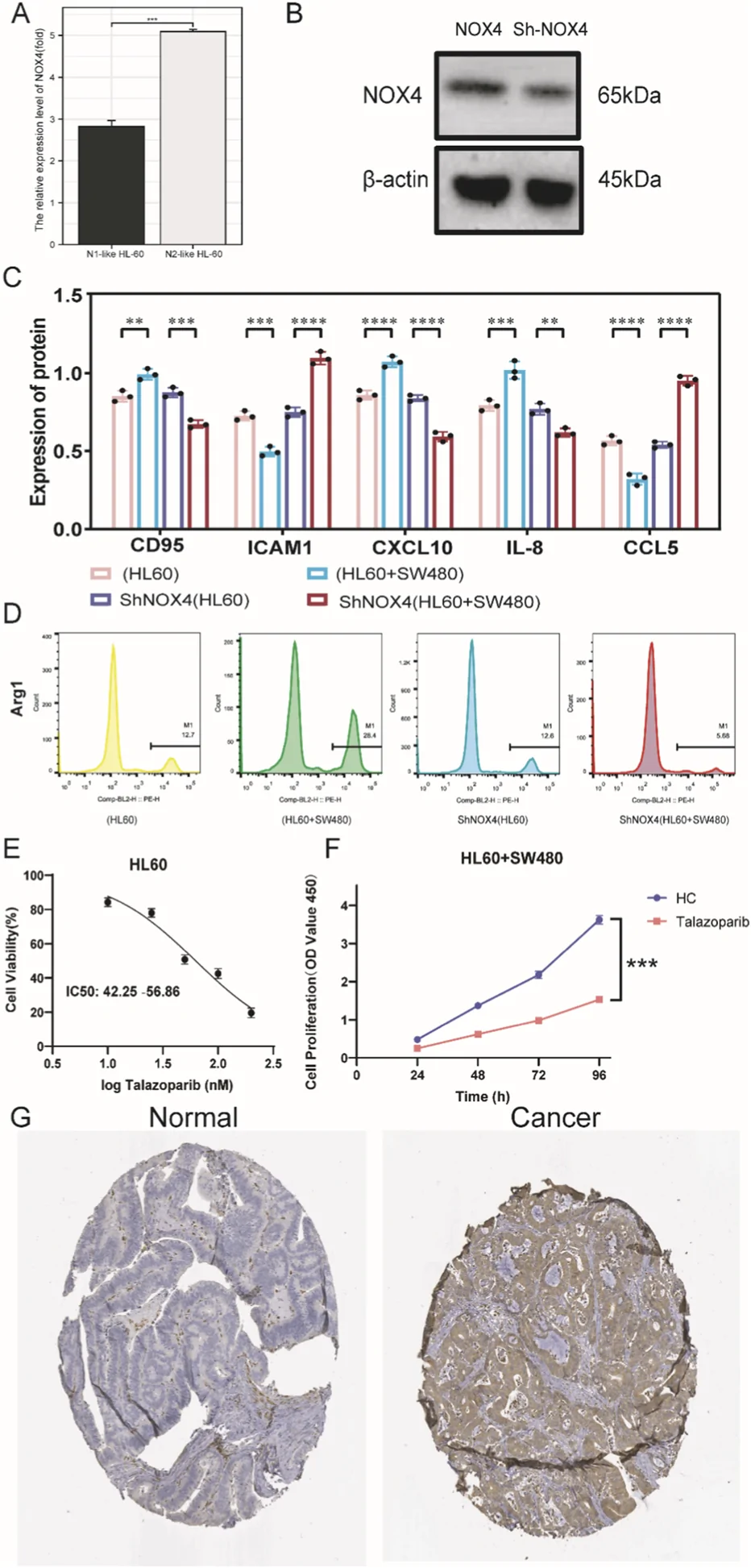
*In vitro* assay examination of the pathogenic role of NOX4 in COAD patients. **(A)** qRT-PCR examination of *NOX4* expression in the HL-60 cell line. **(B)** Western blotting examination of the knockdown efficacy of NOX4. **(C)** N1-like and N2-like biomarker examination in the HL-60 cell line. **(D)** Arg1 flow cytometry examination. **(E)** IC_50_ examination of talazoparib. **(F)** CCK-8 assay examination of the efficacy of talazoparib. **(G)** Representative immunohistochemical images of NOX4 expression in normal colorectal tissue and colorectal cancer tissue retrieved from the Human Protein Atlas (HPA). Images adapted from the Human Protein Atlas under the CC BY 4.0 license.

It is seen that NOX4 was upregulated in the N2-like HL-60 cell line rather than the N1-like HL-60 cell line (Figure 9A). Moreover, IHC examination illustrated that NOX4 expression was upregulated in the COAD samples than normal samples (Figure 9G). Next, following NOX4 knockdown in mature HL-60 cells and supplementation with SW480 cells, we discovered decreased expression of the N2-like and increased expression of the N1-like neutrophil biomarkers (Figures 9B–D). These results highlight the NOX4 COAD pathogenic role in regulating the neutrophil-malignant cell axis. In addition, for talazoparib validation, we treated the HL-60 cell line with an appropriate concentration and collected the supernatant; we found that the supernatant of HL-60 cells treated with talazoparib could restrain the growth of SW480 cancer cells (Figures 9E,F).

## 4 Discussion

COAD remains difficult to treat because patients with similar clinical characteristics can exhibit profoundly different TME states, metabolic programs, and inflammatory dynamics that shape their drug responses and foster adaptive resistance (Zeng et al., 2025a). In this study, we translate succinylation-linked metabolic regulation and neutrophil-driven inflammation into an end-to-end AI-enabled drug design framework that goes beyond prognostic modeling: SN biology is used to define actionable patient strata, while deep-learning-derived subgroups reveal therapy-relevant heterogeneity, and an integrative multi-omics interpretation is used to nominate a druggable hub target with clear immune and redox relevance. Crucially, the workflow connects target identification → candidate prioritization → candidate evaluation using AI-supported ranking together with ADMET-aware filtering and structure-informed assessment, all of which directly match the collective emphasis on accelerating drug discovery, improving efficacy, and designing resistance-aware precision oncology strategies. Rather than presenting a single biomarker, our study provides a reproducible blueprint for how AI can integrate systems biology and structural/ADMET considerations to generate testable therapeutic hypotheses tailored to distinct tumor states, enabling rational repurposing or multitarget strategies that can be experimentally validated and iteratively optimized.

Succinylation or lysine succinylation is a process that regulates the hemostasis of physiological conditions (Shen et al., 2023). In cancer cells, succinylation events are associated with metabolic reprogramming, which contribute to metabolic and immune microenvironment reprogramming (Tong et al., 2021). For COAD patients, research indicates that succinylation-related *PCED1A* can be considered as a hub gene involved in cancer, metabolic reprogramming, immune dysregulation, and prognostic indications. Moreover, neutrophils are innate immune cells that can form NETs to enhance COAD progression (Jiang et al., 2025). The increased proportion of neutrophils in COAD patients is associated with poor prognosis (Zhang et al., 2023). *NOX4* has emerged as a critical gene in the context of cancer, particularly because of its involvement in the generation of ROS and association with CD8^+^ infiltration (Ford et al., 2020). *NOX4* can regulate the M2 tumor-associated macrophages via PI3K–AKT signaling to promote lung cancer growth (Huang et al., 2023). Furthermore, pan-cancer analyses indicate that NOX4 is an emerging biomarker; it can inhibit COAD progression by suppressing ferroptosis (Zhu et al., 2025). Talazoparib is commonly used as a chemotherapy drug for the treatment of breast cancer and has been shown to significantly improve the clinical outcomes of triple-negative breast cancer (Litton et al., 2018). In conclusion, by employing an AI-powered framework with multi-omics studies, we formulated an SN-associated risk stratification and therapeutic strategy. We also identified *NOX4* as an SN-associated hub gene involved in COAD pathogenesis. Significantly, the immune and molecular features of NOX4 were estimated at the bulk and single-cell levels, especially in virtual cells powered by AI. Our study provides additional insights into precision and personalized medicine for COAD patients by identifying NOX4-succinylation-associated molecular patterns in neutrophils in the progression of COAD. However, there remain some limitations to our study. For instance, our predictive and therapeutic model performances should be estimated in large and multi-cohort studies for enhanced robustness. Moreover, NOX4 succinylation mechanisms in neutrophils should be elucidated in preclinical studies. Finally, talazoparib should be evaluated in preclinical and clinical trials to assess its therapeutic accuracy and efficacy.

## Data Availability

The datasets analyzed in this study are publicly available in The Cancer Genome Atlas (TCGA-COAD), the NCBI Gene Expression Omnibus under accession numbers GSE18462, GSE23878, GSE39084, GSE17536, GSE17537, and GSE146771, and the Human Protein Atlas. Any additional data supporting the conclusions of this article will be made available by the authors without undue reservation.
