# Supplementary material for: Artificial-intelligence- and multi-omics-guided predictive and drug repurposing framework construction for colorectal cancer: evidence from succinylation–neutrophil signatures
Source: Front Cell Dev Biol. 2026 Sep 3;14:1808514. doi: 10.3389/fcell.2026.1808514 (PMC13582576; doi:10.3389/fcell.2026.1808514)
Supplement: Supplementary file 1 [file DataSheet1.docx]

**Figure S1: Pre-processing of single-cell level. (A-B)** QC metrics of single-cell data across samples. **(C)** PCA analysis of cells based on cell cycle phase (G1, S, G2M). **(E-G)** Dimensionality reduction analysis.
